# An algorithm for discovering vital nodes in regional networks based on stable path analysis

**DOI:** 10.1038/s41598-023-39174-7

**Published:** 2023-09-16

**Authors:** Yan Liu, Yimin Liu, Fenlin Liu, Jiaxing Fan, Zhiyuan Tao

**Affiliations:** 1 State Key Laboratory of Mathematical Engineering and Advanced Computing, Zhengzhou, 450001 China; 2 Key Laboratory of Cyberspace Situation Awareness of Henan Province, Zhengzhou, 450001 China

**Keywords:** Computational science, Computer science, Information technology, Scientific data, Statistics

## Abstract

Vital node discovery is a hotspot in network topology research. The key is using the Internet’s routing characteristics to remove noisy paths and accurately describe the network topology. In this manuscript, a vital regional routing nodes discovery algorithm based on routing characteristics is proposed. We analyze the stability of multiple rounds of measurement results to overcome the single vantage point’s path deviation. The unstable paths are eliminated from the regional network which is constructed through probing for target area, and the pruned topology is more in line with real routing rules. Finally, we weight the edge based on the actual network’s routing characteristics and discover vital nodes in combination with the weighting degree. Unlike existing algorithms, the proposed algorithm reconstructs the network topology based on communication and transforms unweighted network connections into weighted connections. We can evaluate the node importance in a more realistic network structure. Experiments on the Internet measurement data (275 million probing results collected in 107 days) demonstrate that: the proposed algorithm outperforms four existing typical algorithms. Among 15 groups of comparison in 3 cities, our algorithm found more (or the same number) backbone nodes in 10 groups and found more (or the same number) national backbone nodes in 13 groups.

## Introduction

The expansion of the Internet brings unprecedented pressure to network operation and maintenance (O &M). On account of limited resources, network O &M personnel would pay more attention to the vital nodes in the network to guarantee the network’s quality of service (QoS). Vital nodes discovery can mine out the important routing nodes in the network, help network O &M personnel optimize O &M strategies, improve efficiency, and prevent catastrophic failures. Besides, this work can also provide a reference for optimizing existing network protocols and help the network recover more quickly and efficiently after node failure^1^. Although existing vital nodes discovery research has achieved rich results, the research seldom considers the actual routing situation of the Internet. The routing characteristics of the actual Internet greatly affect the characterization of the vital nodes of regional networks. How to obtain accurate regional network topology is of great practical significance for discovering vital nodes in target areas.

The research on vital nodes discovery originated from graph theory research based on complex networks. Although these methods do not consider the characteristics of the actual Internet, they still have guiding significance for existing research. Methods in this category include Degree Centrality (DC)^2^, Clustering Coefficient^3^, K-shell Decomposition^4^, Closeness Centrality (CC)^5^, Betweenness Centrality (BC)^6^.In addition, some new methods have also been proposed recently. Xu et al.^7^ proposed a new node-centring method called unsigned Laplacian feature vector centring, considering the mutual influence between nodes and their incident edges. Ullah et al.^8^ provided a Local-and-Global-Centrality (LGC) measuring algorithm to identify the vital nodes through handling local as well as global topological aspects of a network simultaneously. To address the issues of low accuracy and high complexity in traditional online social networks(OSNs), Luo et al.^9^ built a relationship matrix resolving model to identify vital nodes by complying with community, which is capable of effectively identifying influential nodes in the network. Li et al.^10^ presented a novel local centrality to identify vital nodes by combining the influence of the node itself and neighbor as well as clustering coefficient information. Rezaei et al.^11^ proposed a data-driven vital node identification method based on machine learning to address the weak adaptability of heuristic methods based on mathematical expressions.

In recent years, some researchers have optimized the computational process based on such methods to deal with large-scale data. Matteo et al.^12^ presented two randomized algorithms. Michele et al.^13^ used an adaptive sampling technique to sample the data in the topology graph to reduce the calculation cost of BC and approximated the BC of vital nodes in the original topology on this basic. Dong et al.^14^ proposed a localized strategy that can find vital nodes without global knowledge of the network. Sunil^15^ provided a GNN-based (Graph Neural Network) inductive framework to approximate BC using the message passing mechanism. These methods are almost all based on the macro-statistical characteristics of graphs^16^, and pay less attention to the routing characteristics of the actual Internet.

Unlike the above methods, another research takes certain characteristics of the actual Internet into account. Ulrik et al.^17^ proposed Traffic Load Centrality (TLC). TLC simulates the transmission process of network data packets, and only uses the transmission on the shortest path to describe the load carried by the node, which is used to describe the node’s importance. Linton et al.^18^ proposed Flow Betweenness Centrality (FBC) by considering the shortest path and the non-shortest paths at the same time. FBC believes that the larger the proportion of paths passing through a node among all the non-repeated paths in the network, the more critical the node is. Shlomi et al.^19^ combined FBC and network routing and proposed the Routing Betweenness Centrality (RBC). They assumed that the routing table is known and mined vital nodes according to the number of paths connected by the target node. Leonardo et al.^20^ proposed the Load Centrality (LC), which mined vital nodes in the network by calculating the expected load on the routing nodes. Alain et al.^21^ considered the heterogeneity of edges between nodes in real-world networks and introduced the Weighted Degree Centrality to measure the importance of nodes. To address the issue of low-degree nodes tending to have higher clustering coefficients, Xuefei et al.^22^ proposed the Weighted Clustering Coefficient to assess top-k key nodes by taking into account both the node’s clustering coefficient and its degree. This kind of methods regulate the characteristics of the network such as path, traffic and protocol to a certain extent, and mine the vital nodes on this basis. These methods solve some phenomena on the Internet, but they are still difficult to adapt to the actual network.

In view of the above problems and the difficulty of obtaining the Internet routing tables, this manuscript proposes an algorithm for discovering vital nodes in regional networks based on stable path analysis. The main idea is to obtain stable paths from the vantage points to the target based on a large number of repeated and long-term probing, and the vital nodes is discovered based on statistical theory. First, we deploy vantage points inside and outside the target area, and the path information between nodes of the target area is obtained through Internet measurement. On this basis, a preliminary topology graph is constructed. Second, we extract the stable paths of the target network from measured path information and eliminate the unstable paths to denoise the constructed preliminary network topology. Finally, we weight the edges according to the number of stable paths passing through adjacent nodes, and rank the nodes according to the weighting results. The main contributions of this manuscript are the following:

- We propose a network topology denoising method based on stable paths. This method can effectively reduce the data processing scale and reveal the role of stable paths in actual networks.
- We combine the edge-weighting method with stable paths, which can accurately describe the role of edges between nodes.
- Experiments on the Internet measurement data (275 million probing results collected in 107 days) of Chengdu, Zhengzhou, and Hangzhou in China demonstrate that: Compared with the classical algorithms (Degree Centrality, Betweenness Centrality, Weighted Degree Centrality, Routing Betweenness Centrality), the proposed algorithm can better describe the importance of nodes in the target area and can find more accurate backbone nodes.

The structure of this manuscript is organized as follows. In section Vital nodes discovery algorithm in regional networks based on stable path analysis, we give the details of the proposed algorithm and its main steps. In section Algorithm analysis, we analyze the effect of the proposed algorithm in principle. In section Experiments, we perform experimental evaluations to quantify the benefits of our algorithm and discusses the results. Section Conclusion concludes the whole manuscript.

## Vital nodes discovery algorithm in regional networks based on stable path analysis

**Figure 1:**
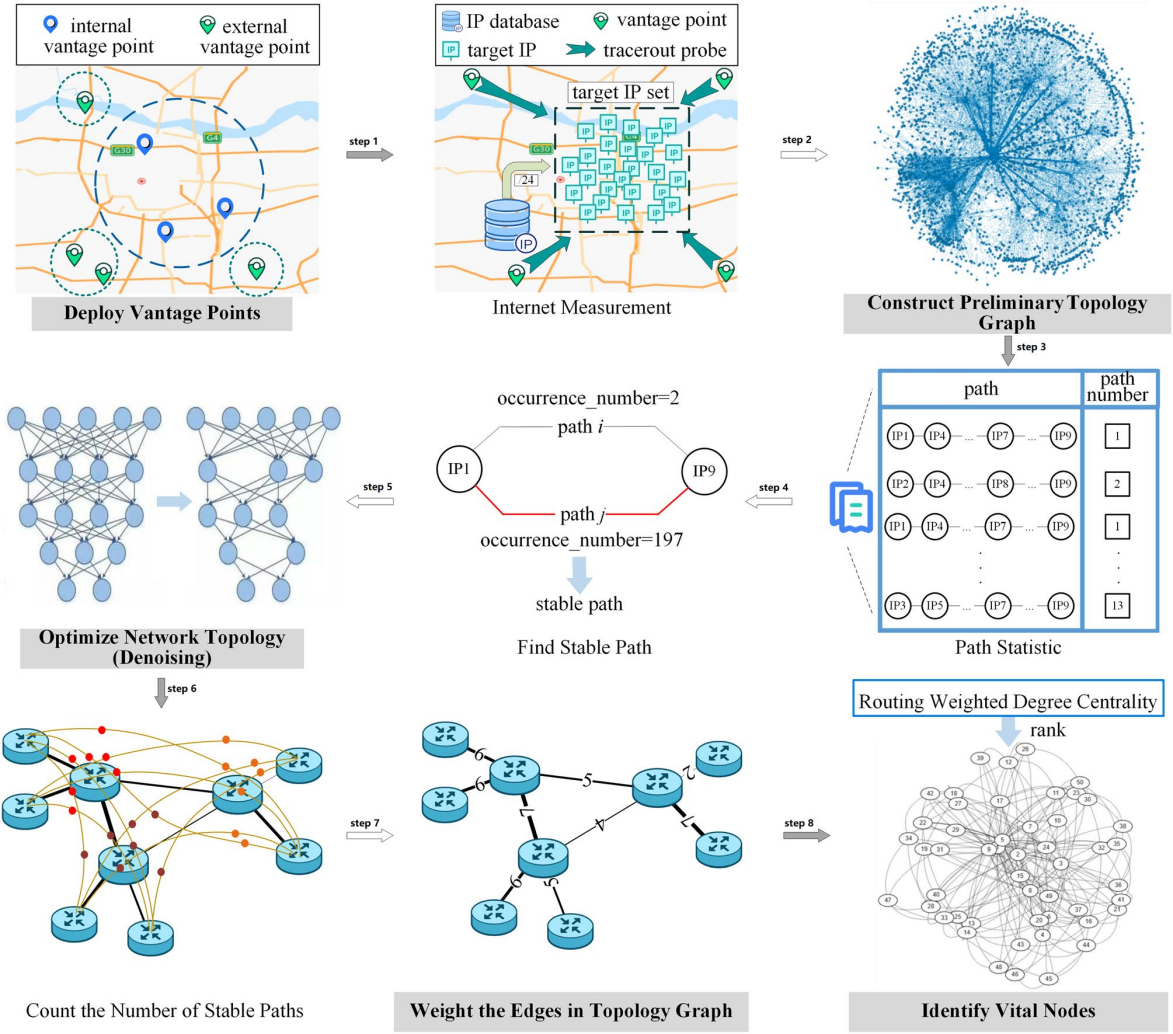
Overall architecture of proposed algorithm.

The communication among nodes on the Internet is determined by routing rules, which are difficult to obtain directly. However, these routing rules can be approximated by a large number of repeated probes and statistical analyses. In addition, there is a large amount of path information in the massive data measured. The path information contains stable paths determined by the routing rules. This is similar to travel planning on the highway in real life, the planned route is fixed when there is no congestion. Therefore, it is possible to obtain a stable path from the vantage point to the target through Internet measurement and construct a network topology composed of only stable paths. Based on this idea, this section proposes an algorithm for discovering vital nodes in regional networks based on stable path analysis. The proposed algorithm is based on stability analysis of multi-round measurement results to overcome path deviation caused by a single measurement, eliminate unstable paths in the network, and obtain a regional network topology that is more in line with real routing rules. Unlike existing algorithms, this algorithm reconstructs the network topology based on traffic, transforming the unweighted internet into a weighted connection, and studying node importance assessment in a structure closer to the actual internet. This can effectively overcome the inapplicability of traditional algorithms on non-cooperative networks whose size, node relationships and routing rules are almost unknown to us. Figure 1 illustrates its overall architecture.

The main steps of the algorithm are as follows.

*Step 1: Deploy the vantage points.* When only a single vantage point is used to probe the target IP, the measurement results are prone to spatial offset and accidental. Therefore, a set of vantage points $\textit{V}_{V}$ is selected, including $n_{I}$ vantage points located inside the target area *A*, and $n_{O}$ vantage points located outside the target area.

*Step 2: Acquire the preliminary topology of the target area.* Firstly, retrieve the IP address segments $\textit{S}_{A}$ assigned to target area *A* from databases *D* such as IPIP, WHOIS, and IP2location (detailed in section Experimental setup), and obtain more accurate IP address segments by intersecting the address segments from multiple data sources. Then, enumerate the IP address in each IP address segment to form the target IP set $\textit{V}_{T}$. Finally, use the vantage point set $\textit{V}_{V}$ to measure the target IP set $\textit{V}_{T}$ with multi-rounds, continuous and high-frequency Internet measurement, and acquire the network topology information such as paths and delays. According to the measurement results, the node set $\textit{V}_{A}$ and the edge set $\textit{E}_{A}$ located in the target area are extracted, and the preliminary topology *G* is constructed.

*Step 3: Optimize the network topology based on stable paths.* Count the paths, and find the stable path $\textit{P}_{S}$ in the network according to the routing rules. Then, eliminate the unstable path in the topology to optimize the topology, and obtain the topology $G_S$ that only retains stable paths after denoising.

*Step 4: Weight edges based on routing characteristics.* By applying formula (5) to weight the edges of the denoised topology, we can obtain a weighted topology of the target area. The weights represent the actual traffic carried by the edges.

*Step 5: Identify the vital nodes in the regional network.* Calculate the routing weighted degree centrality (RWDC) of each node in the topological graph, and rank the nodes according to RWDC, then identify the vital nodes in the regional network. The calculation of the Routing Weighted Degree Centrality for node $v_{}$ is shown in formula (1).

1 $$\begin{aligned} \textit{RWDC}(v_{i})= \sum _{v_{j}\in \mathbf {V_{i}}}{\frac{w(e_{i,j})}{card(\mathbf {P_{G})}}}=\frac{\sum _{v_{j} \in \mathbf {V_{i}}} \sum _{p \in \mathbf {P_{G}}} {\delta (p,i,j)}}{card(\mathbf {P_{G})}} \end{aligned}$$

$\mathbf {V_{i}}$ is the neighbour set of node $v_{i}$, $\mathbf {P_{G}}$ is the set of stable paths obtained by measuring the topology graph *G*, $card(\mathbf {P_{G}})$ is the number of elements in set $\mathbf {P_{G}}$, $w(e_{i,j})$ and $\delta (p,i,j)$ are defined in equations (5) and (6). The process of the proposed algorithm is outlined in Algorithm 1. The algorithm first utilizes the probe source $V_{v}$ to continuously and high-frequency measure the target area *A* and extracts the nodes and edges of target area *A* based on the measurement paths (a path from the probe source to the target node) to construct the initial topology structure of target area *A* (lines 1-5). Then, count the number of occurrences of all measurement paths and retain the path with the highest number of occurrences as a stable path to construct a denoised graph $G_{s}$ (lines 6-16). Afterwards, the number of times each edge in $G_{s}$ appears in the stable path is counted as the weight of the edge to obtain a weighted graph (lines 17-22). Finally, calculate the RWDC value of each node in $G_{w}$ and sort them in descending order (lines 23-26). Our method is similar to BC, but it differs greatly from BC. Firstly, BC assumes a unit traffic flow between all node pairs, our method is based on actual traffic routing demand. It does not require traffic to only be transmitted on the shortest paths between nodes. Secondly, our method discards traffic between a node pair when that traffic follows a path that does not have the highest occurrence, achieving denoising of the topology and reducing the impact of path offset on evaluating the importance of nodes. Finally, BC applies a static calculation of traffic load per link based on the unit traffic flow between all node pairs, our method weights the edges by determining the traffic load per link based on measurements and converts the edge weight into node importance through formula (1). In our algorithm, the topology denoising method based on stable paths and the edge-weighting method based on routing characteristics are the most critical parts. We discuss the implementation processes in detail.


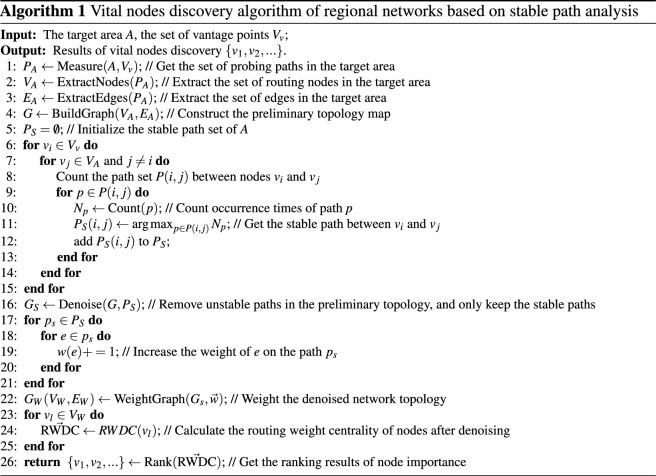


### The topology denoising method based on stable paths

Among the paths between node $\textit{v}_{i}$ and node $\textit{v}_{j}$ (from vantage point to target IP), the path with the most occurrence times is regarded as the stable path. The path $\textit{P}_{n}$ between $\textit{v}_{i}$ and $\textit{v}_{j}$ is denoted as:

2 $$\begin{aligned} \textit{P}_n= \left\{ v_{i},...,v_{m},...,v_{j}\right\} \end{aligned}$$

where $v_{m}$ represents a node in the path $\textit{P}_{n}$.

The set of paths between $\textit{v}_{i}$ and $\textit{v}_{j}$ obtained by *N* times probing in time *t* is denoted as $\textit{P}(i,j)$:

3 $$\begin{aligned} \textit{P}(i,j)= \left\{ \textit{P}_{1},\textit{P}_{2},...,\textit{P}_{n},...,\textit{P}_{N}\right\} , n\in [1,N] \end{aligned}$$

The occurrence times of path *p* is denoted as $\textit{N}_{p}$. Then the stable path set $\textit{P}_{S}(i,j)$ between $\textit{v}_{i}$ and $\textit{v}_{j}$ is:

4 $$\begin{aligned} \textit{P}_{S}(i,j) = \mathop {\arg \max }_{p}N_{p}, p\in \textit{P}(i,j) \end{aligned}$$

For ease of understanding, this section describes the process of denoising network topology based on stable paths with the following example.

**Figure 2:**
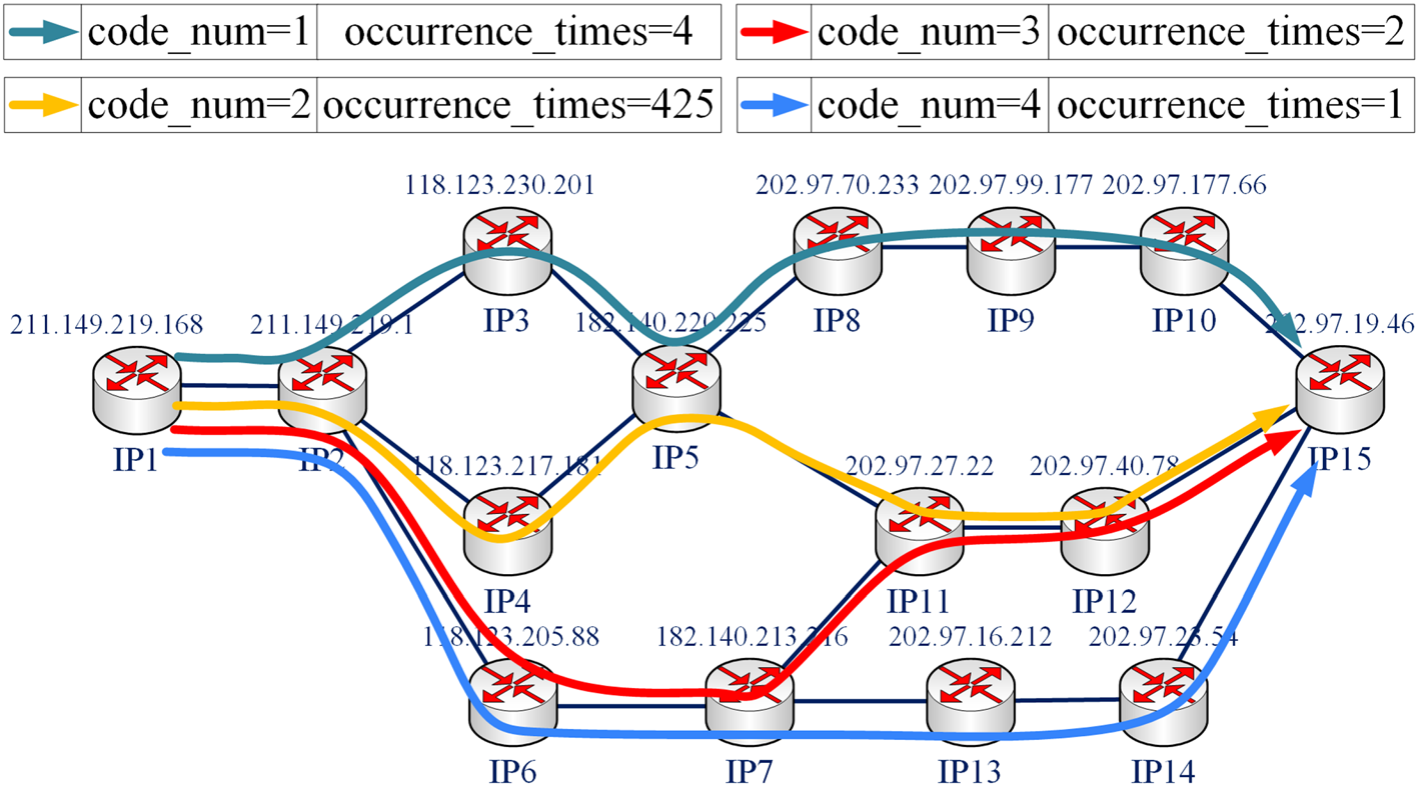
An example diagram for the proposed topology denoising method.

As shown in Fig. 2, 432 completed paths can be extracted from the measurement results of $\textrm{IP}_{1}$ (211.149.219.168) to $\textrm{IP}_{11}$ (202.97.19.46), including 4 different types of paths:

Path 1: $$\textrm{IP}_{1}-\textrm{IP}_{2}-\textrm{IP}_{3}-\textrm{IP}_{5}-\textrm{IP}_{8}-\textrm{IP}_{9}-\textrm{IP}_{10}-\textrm{IP}_{15}$$, occurence_times = 4

Path 2: $$\textrm{IP}_{1}-\textrm{IP}_{2}-\textrm{IP}_{4}-\textrm{IP}_{5}-\textrm{IP}_{11}-\textrm{IP}_{12}-\textrm{IP}_{15}$$, occurence_times = 425

Path 3: $$\textrm{IP}_{1}-\textrm{IP}_{2}-\textrm{IP}_{6}-\textrm{IP}_{7}-\textrm{IP}_{11}-\textrm{IP}_{12}-\textrm{IP}_{15}$$, occurence_times = 2

Path 4: $$\textrm{IP}_{1}-\textrm{IP}_{2}-\textrm{IP}_{6}-\textrm{IP}_{7}-\textrm{IP}_{13}-\textrm{IP}_{14}-\textrm{IP}_{15}$$, occurence_times = 1

The path with the most occurrence times is Path 2, which accounted for 98.38% of the total number of paths. Therefore, Path 2 is the stable path from the vantage point to $\textrm{IP}_{15}$ (202.97.19.46), and the stable path proportion is 0.9838. When denoising network topology, delete Path 1, Path 3, Path 4 and keep Path 2 only. Besides, if two paths have the same occurrence times and both are the most frequent paths, then both paths are regarded as stable paths.

### The edge-weighting method based on routing characteristics

The proposed method takes the number of stable paths passing through an edge as the weight of the edge. If all stable paths in the network are denoted as $\textit{P}_{S}$, the calculation formula for the weight $w(e_{g,k})$ of the edge $e_{g,k}$ between two adjacent nodes $\textit{v}_{g}$ and $\textit{v}_{k}$ is as follows:

5 $$\begin{aligned} w(e_{g,k}) = \sum _{p\in \textit{P}_{S}} \delta (p,g,k) \end{aligned}$$

If the path *p* in $\textit{P}_{S}$ passes the edge $e_{g,k}$, then $\delta (p,g,k)=1$, otherwise $\delta (p,g,k)=0$. The definition of $\delta (p,g,k)$ is:

6 $$\begin{aligned} \delta (p,g,k) = {\left\{ \begin{array}{ll} 1 &{} v_{g}\in \textit{V}_{p} \ and \ v_{k}\in \textit{V}_{p}\\ 0 &{} v_{g}\notin \textit{V}_{p} \ or \ v_{k}\notin \textit{V}_{p} \end{array}\right. } \end{aligned}$$

where, $\textit{V}_{p}$ is the set of all nodes on path *p*.

**Figure 3:**
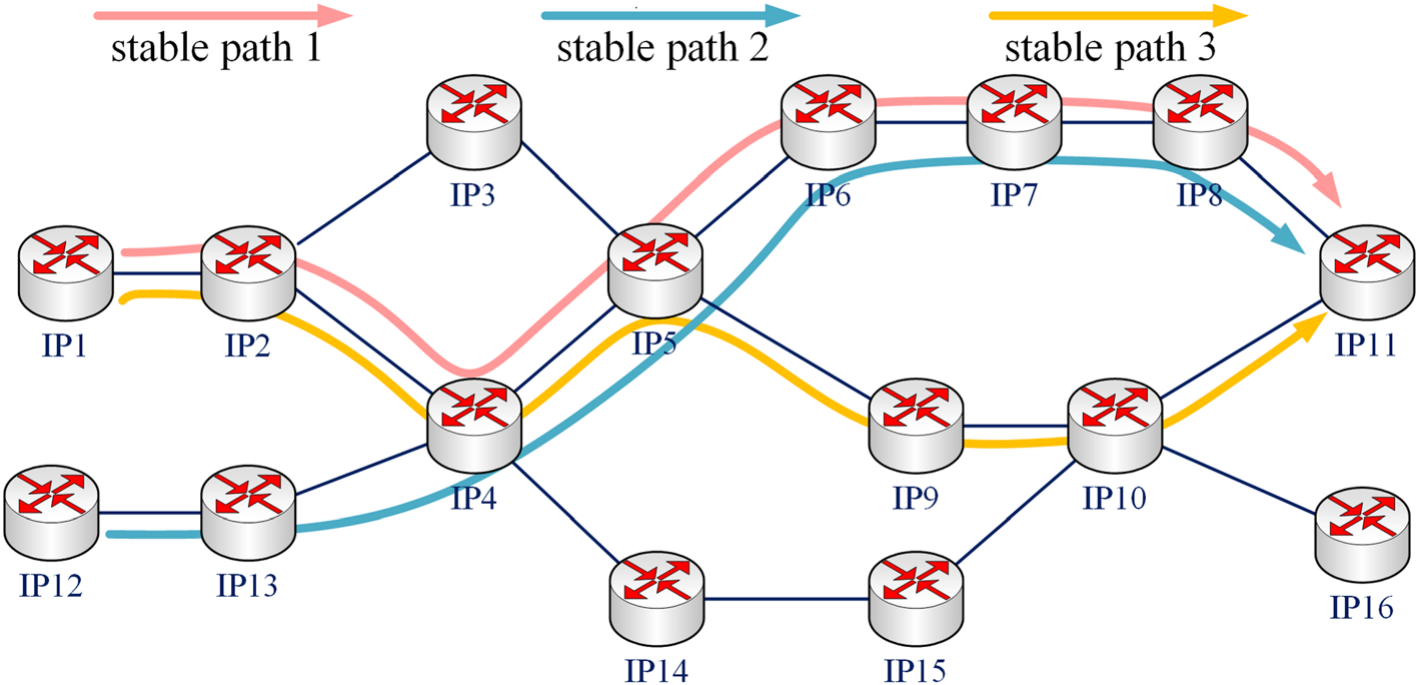
An example diagram for the proposed edge-weighting method.

For ease of understanding, this section describes the method of edge-weighting based on routing characteristics with the example in Fig. 3.

As shown in Fig. 3, in actual communication, there are 3 stable paths passing the edge ($\textrm{IP}_{4}$-$\textrm{IP}_{5}$):

Stable path 1: $$\textrm{IP}_{1}-\textrm{IP}_{2}-\textrm{IP}_{4}-\textrm{IP}_{5}-\textrm{IP}_{9}-\textrm{IP}_{10}-\textrm{IP}_{11}$$

Stable path 2: $$\textrm{IP}_{12}-\textrm{IP}_{13}-\textrm{IP}_{4}-\textrm{IP}_{5}-\textrm{IP}_{6}-\textrm{IP}_{7}-\textrm{IP}_{8}-\textrm{IP}_{11}$$

Stable path 3: $$\textrm{IP}_{1}-\textrm{IP}_{2}-\textrm{IP}_{4}-\textrm{IP}_{5}-\textrm{IP}_{6}-\textrm{IP}_{7}-\textrm{IP}_{8}-\textrm{IP}_{11}$$

So the weight of edge ($\textrm{IP}_{4}-\textrm{IP}_{5}$) is $w(e_{4,5} )=3$.

## Algorithm analysis

In the algorithm proposed, the topology denoising based on stable paths and the edge-weighting method based on routing characteristics are the most important steps, and its effectiveness will be analyzed in this section. Accurate topological characterization is significant to solve the problem of vital nodes discovery. The proposed algorithm can eliminate edges that have a negative impact on vital nodes discovery, and weight edges between nodes more accurately. Therefore, it can accurately reflect the topological characteristics of the regional network.

### Analysis of the topology denoising method based on stable paths

When conducting research on vital nodes discovery, it is necessary to consider the amount of communication carried by nodes and the amount of transmission on edges between nodes. There are two kinds of edges: edges on fixed and non-fixed paths. On the one hand, communication protocols are often designed based on ideal conditions at the beginning, without considering the unstable path. On the other hand, the appearance of the unstable path is due to network congestion, which is caused by many reasons, so it is difficult to consider the importance of nodes based on unstable path. Therefore, in the process of discovering vital nodes, the research should only be based on stable paths, and eliminate noise data such as unstable paths.

Existing research on vital nodes discovery usually add all existing nodes and edges to the network topology graph. By analyzing the measurement results, it comes to a conclusion: there exist stable paths for communication between nodes on the Internet. Therefore, the proposed algorithm denoises the topology graph based on the stable path and only the stable path in the actual communication is retained in the final network topology graph.

**Figure 4:**
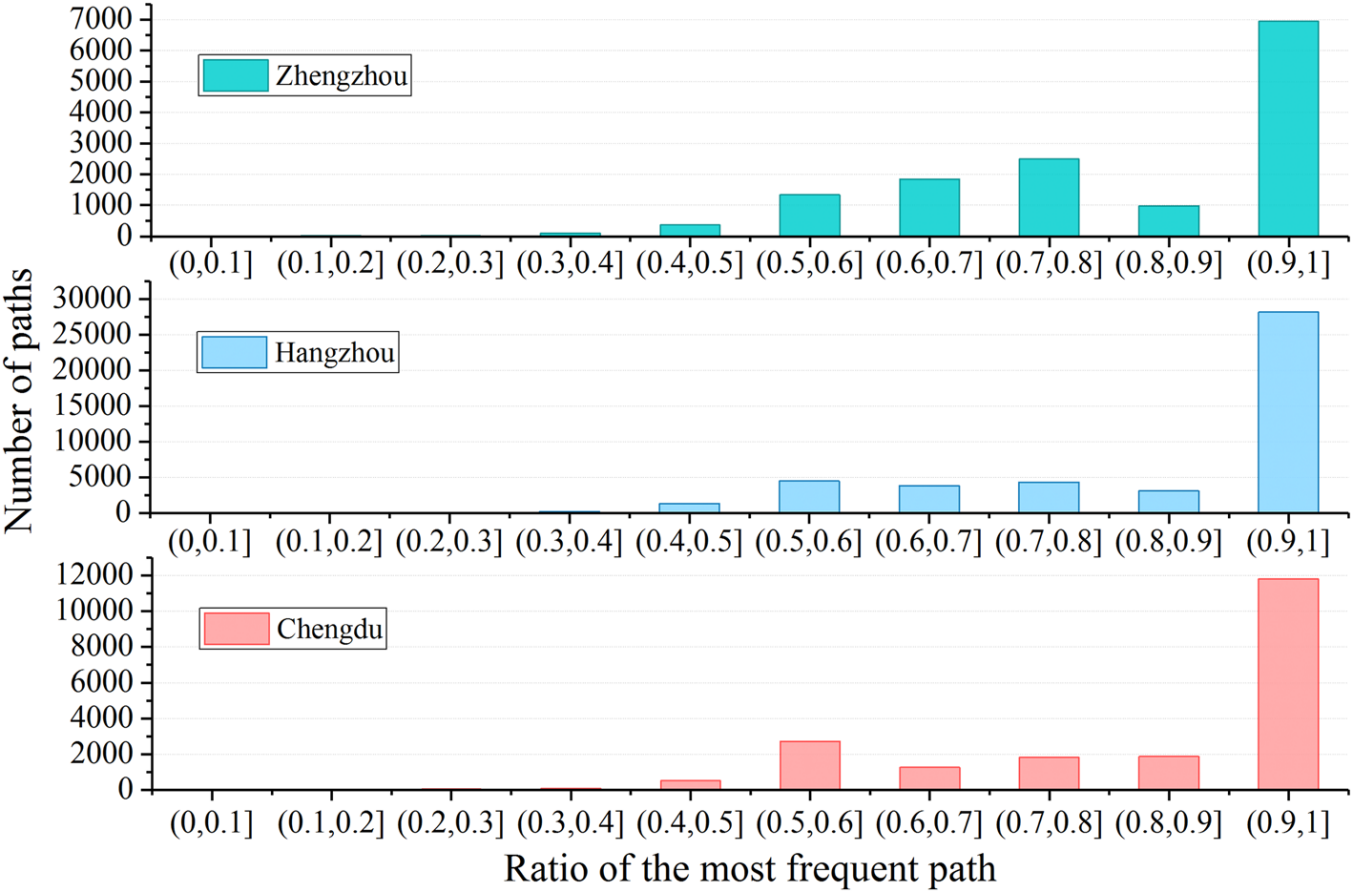
Path proportion statistics for target IP.

Among the results of 40-day Internet measurements in the three cities, there are 28,987,966 responses, including 168,594 different paths, and 79,166 are stable paths. For the completed measurement results in the three cities, the path with the highest proportion of occurrence times in the total path occurrence times is counted respectively. The results are shown in Fig. 4.

In Fig. 4, the x-axis represents the ratio of the most frequent path’s occurrence times to the total number of paths to a single IP; the y-axis represents the number of paths in the interval. As shown in Fig. 4, in the measurement results of Zhengzhou, Hangzhou and Chengdu, the proportion of major paths to the target node is basically more than 50%, and these paths are called stable paths. As shown in Table 1, the proportions of stable paths in the measurement results are 83.1%, 86.1%, and 85.5%, respectively. This indicates that there is indeed a stable path in actual network communication.

**Table 1:** The ratio of occurrence times of stable paths in all paths.

City	Number of stable paths	Occurrence times of stable paths	Occurrence times of all paths	Ratio of occurrence times of stable paths
Zhengzhou	14,048	4,557,929	5,484,648	83.1%
Hangzhou	45,108	12,911,231	14,977,107	86.1%
Chengdu	20,010	7,296,142	8,526,211	85.5%
Total	79,166	24,765,302	28,987,966	85.4%

Due to the limited network resource, network O &M personnel need to conduct hierarchical management of router nodes to ensure the network’s QoS. From the perspective of routing characteristics, because of the existence of load balancing and other strategies, some communications will not pass through stable paths. The existence of these paths is the noise data in the process of vital nodes discovery. Taking the highway as an example for analogy, when the road conditions are good, the driver will choose the optimal one; but when congestion occurs, the driver will choose the sub-optimal way to avoid the congestion. Obviously, the nodes on the optimal path are the actual vital nodes. Routing rules determine the existence of stable paths. Therefore, the network topology denoising method based on stable paths proposed in this manuscript reduces the data size, enhances the ability to process data, reduces the interference caused by load balancing, improves the efficiency of vital nodes discovery, and can also obtain more accurate vital nodes discovery results.

Take the data of the first 40 days in Chengdu as an example to compare the network scale before and after denoising, as shown in Table 2.

**Table 2:** Comparison of network scale before and after denoising.

	Preliminary topology graph	Denoised topology graph
Number of nodes	9710	9336
Number of edges	127,205	56,685
Number of paths	43,944	20,010

As can be seen from Table 2, denoising the network can reduce about 3.8% of nodes, 55.4% of edges, and 54.4% of paths. The reduction of a small number of nodes is caused by the deployment of vantage points and load balancing. These nodes are not on the stable path from the selected vantage point, so they will be removed during the topology optimization process. This process will have a certain impact on the coverage of vital nodes, but has no effect on the accuracy of vital nodes discovery. If we want to increase the coverage of vital nodes, we can select different combinations of vantage points to measure the target network separately.

The existing research object of vital nodes discovery is usually static network models which do not consider the transmission of traffic in the network, or simply assume that traffic is transmitted equally on the edges. However, due to the existence of routing rules, the number of paths passing the edges between different nodes-in-pairs is significantly different. So these edges have great differences in the traffic they carry and the roles they play. Therefore, their influence on the connected nodes is also different. In this case, this manuscript weights edges based on stable paths in actual communication, and then combines the weights of edges to evaluate the importance of nodes to obtain more accurate results of vital nodes discovery.

**Figure 5:**
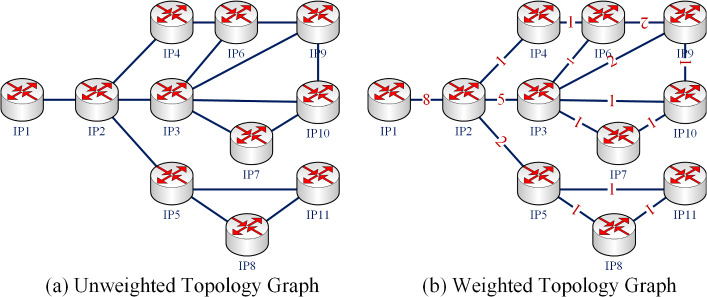
Comparison of the unweighted and weighted topology graph.

Take Fig. 5 as an example to illustrate the necessity of constructing a weighted topology graph.

Suppose the paths existing in the communication from $\textrm{IP}_{1}$ to $\textrm{IP}_{9}$, $\textrm{IP}_{10}$ and $\textrm{IP}_{11}$ are:

$$\textrm{IP}_{9}\ : [\textrm{IP}_{1}-\textrm{IP}_{2}-\textrm{IP}_{4}-\textrm{IP}_{6}-\textrm{IP}_{9}]$$, $$[\textrm{IP}_{1}-\textrm{IP}_{2}-\textrm{IP}_{3}-\textrm{IP}_{6}-\textrm{IP}_{9}]$$, $$[\textrm{IP}_{1}-\textrm{IP}_{2}-\textrm{IP}_{3}-\textrm{IP}_{9}]$$

$$\textrm{IP}_{10}: [\textrm{IP}_{1}-\textrm{IP}_{2}-\textrm{IP}_{3}-\textrm{IP}_{10}]$$, $$[\textrm{IP}_{1}-\textrm{IP}_{2}-\textrm{IP}_{3}-\textrm{IP}_{7}-\textrm{IP}_{10}]$$, $$[\textrm{IP}_{1}-\textrm{IP}_{2}-\textrm{IP}_{3}-\textrm{IP}_{9}-\textrm{IP}_{10}]$$

$$\textrm{IP}_{11}: [\textrm{IP}_{1}-\textrm{IP}_{2}-\textrm{IP}_{5}-\textrm{IP}_{11}]$$, $$[\textrm{IP}_{1}-\textrm{IP}_{2}-\textrm{IP}_{5}-\textrm{IP}_{8}-\textrm{IP}_{11}]$$

The unweighted topology graph can be constructed from the above paths, as shown in Fig. 5a. The proposed method weights edges according to the number of paths passing through an edge and the weighted topology graph is constructed, as shown in Fig. 5b.

Calculate the degree centrality (DC) of all nodes in Fig. 5a. The results are shown in Table 3.

**Table 3:** Result of nodes ranking by DC in the unweighted graph.

Node	$IP _{3}$	$IP _{2}$	$IP _{5}$	$IP _{6}$	$IP _{9}$	$IP _{10}$	$IP _{4}$	$IP _{7}$	$IP _{8}$	$IP _{11}$	$IP _{1}$
Degree	5	4	3	3	3	3	2	2	2	2	1

Consider the weight of the edge and calculate the weighted degree centrality (WDC) of all nodes in Fig. 5b. The results are shown in the Table 4.

**Table 4:** Result of nodes ranking by WDC in the weighted graph.

Node	$IP _{2}$	$IP _{3}$	$IP _{1}$	$IP _{9}$	$IP _{5}$	$IP _{6}$	$IP _{10}$	$IP _{4}$	$IP _{7}$	$IP _{8}$	$IP _{11}$
Degree	16	10	8	5	4	4	3	2	2	2	2

**Figure 6:**
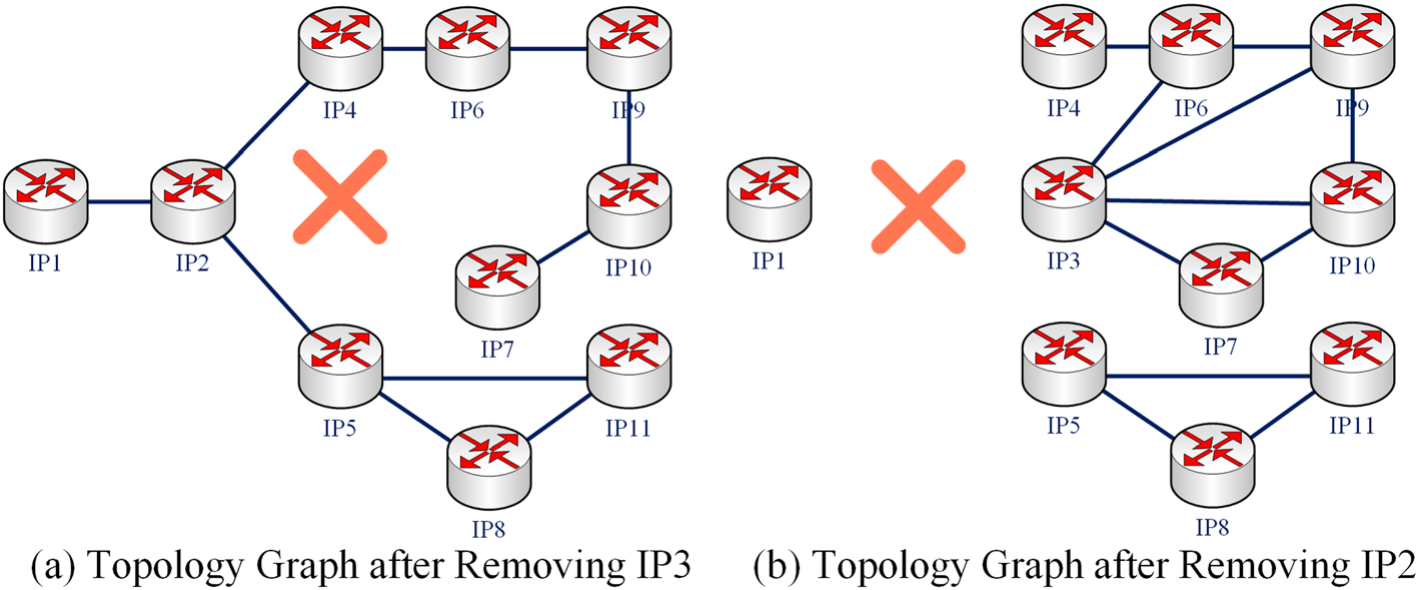
Comparison of the topology graph after removing $IP _{2}$ and $IP _{3}$.

As can be seen from Tables 3 and 4, $IP _{3}$ ranks higher than $IP _{2}$ in the unweighted graph, that is to say, $IP _{3}$ is more important than $IP _{2}$. However, in the weighted graph, $IP _{2}$ ranks higher than $IP _{3}$; that is to say, $IP _{2}$ is more important than $IP _{3}$. In the internet communication process, the arrival of $IP _{3}$ must go through $IP _{2}$. Use the nodes deletion method to evaluate their importance. After removing $IP _{2}$ and $IP _{3}$, respectively, the topology graph of the network is shown in Fig. 6. Obviously, after removing $IP _{3}$, the remaining nodes in the network can still communicate with each other. However, after removing $IP _{2}$, many nodes cannot communicate with each other normally. So $IP _{2}$ plays a more critical role in the network than $IP _{3}$. It can be seen that more accurate ranking results can be obtained by using weighted network topology.

In the existing research, the mining of vital nodes based on the weighting method are not based on the actual topology data, they are still the mining of the mathematical characteristics of the known topology. The proposed algorithm starts with the actual data, and the proposed weighting method is closer to actual network characteristics, which can better reflect the importance of different edges in the network.

## Experiments

In order to verify the feasibility and effectiveness of the proposed algorithm, this section conducts the vital nodes discovery experiment. In the case of obtaining the actual communication paths between all nodes-in-pairs in the target area, we can get the most accurate results of vital nodes discovery. However, this requires deploying a probe at each node in the target network, which is difficult for a medium-sized city. Therefore, this section selects some vantage points to carry out continuous probing (last 107 days) on the IP addresses of the target area. The measurement results could approximate the communication of actual networks. The experimental results show that the performance of the proposed algorithm is better than existing algorithms, indicating that the approximation method is reasonable.

### Experimental setup

Experimental setup in the data acquisition stage are shown in Table 5.

**Table 5:** Experimental setup for data acquisition.

Parameter	Setup
*A*	Zhengzhou, Hangzhou, Chengdu
*D*	Maxmind$^{1}$, IP2location$^{2}$, Whois$^{3}$, IPIP$^{4}$, IPPlus$^{5}$, IPcn$^{6}$
*V*	211.149.219.168, 47.110.233.88, 122.114.14.202
*T*	2 hours

1 http://www.maxmind.com/

2 http://www.ip2location.com/

3 http://www.whois.com/

4 http://www.ipip.net/

5 https://www.ipplus360.com/

6 http://www.ip.cn/.

In Table 5, *A* represents the target areas, *D* represents the IP address databases, *V* represents vantage points, and *T* represents the cycle of probing.

Considering the realistic conditions, this section chooses three cities in China, including Chengdu, Sichuan Province, Hangzhou, Zhejiang Province, and Zhengzhou, Henan Province as the target areas. Then, select the IP address blocks located in the target areas from 6 IP address databases, retain the IP blocks that have appeared in at least 3 IP address databases to form the IP block set $S_{A}$ of the target area.

The real subnet structure and division method are difficult to obtain directly, so this section extracts IP addresses from network segments for probing. IPes in the same network segment are often similar in routing strategy, geographical location and other settings, and often belong to the same organization^23–25^. Based on this situation, this section selects one IP from each /24 IP block to construct the target set $\textit{V}_{T}$. Then, probe $\textit{V}_{T}$ with vantage points at *V* (*V* is composed of three vantage points located in Zhengzhou, Hangzhou and Chengdu).

### Internet measurement

This section uses Scamper^26^ developed by CAIDA for Internet measurement. The IP address blocks of the three target cities were selected from 6 IP address databases, including IPIP, Whois, IPPlus, IP2location, Maxmind and IPcn released in November 2019.

There are 12,748,117 IP addresses in the three target cities, and the three target IP sets contain 60,337 target IP addresses in total. The number of IP addresses and target IP addresses of the three cities are shown in Table 6. In 2019-2020, we probed the target IP addresses in the three cities, obtaining 275,893,827 results in total. The number of /24 blocks covered by the measurement results, and the number of routing nodes and paths extracted from the results are shown in Table 6.

**Table 6:** Statistics of the dataset in Internet measurement.

Target City	# IP addresses	# Target IP addresses	# /24 blocks	# Routing nodes	# Paths
Zhengzhou	2,725,327	11,598	5466	7331	76,014
Hangzhou	7,501,838	30,694	16,443	20,401	248,298
Chengdu	4,2747,36	18,045	7462	10,956	109,568

Due to the unique situation of the layered architecture of China’s Internet, the communication between Internet Service Providers (ISP) without interconnection needs to be forwarded through Internet Exchange Points (IXP) deployed in specific cities. Therefore, in order to avoid the interference of cross-city data, this section only selects a single operator for experimentation. This manuscript uses the data of China Telecom in the above data set as an example to conduct the following vital nodes discovery experiments.

### Results of vital nodes discovery experiment

After obtaining the topological data of target cities, the weighted network topology graph could be constructed and denoised based on the stable paths to obtain the routing weighted degree centrality (RWDC) of the nodes. This section conducted the following three experiments: Experiment on the effect of different Internet measurement durations on the algorithm’s performance, comparison experiment of nodes discovery before and after denoising, and comparison experiment of the proposed algorithm and baseline algorithms. The experimental results are validated according to the existing database.

Notations used in this section are listed in Table 7.

**Table 7:** Symbol definition.

Notations	Descriptions
#	The number of tokens.
Top-*k*	Backbone nodes covered by top *k* nodes in the node importance ranking result.
$B_{N}$	National-level backbone node.
$B_{P}$	Provincial-level backbone node.
*B*	Backbone node, including national-level and provincial-level backbone node.
$\times$	Other node.
*L*	The label of the routing node in public database, including $B_{N}$, $B_{P}$ and *B*.

#### Effect of different durations on the performance of the algorithm

This section compares the network size and ranking results on measurement results collected in 5 days (60 rounds), 40 days (360 rounds) and 107 days (1,284 rounds), respectively. Take Chengdu as an example to show the results, as shown in Tables 8 and 9.

**Table 8:** Comparison of the data scale collected in 5 days, 40 days and 107 days.

	5 Days		40 Days		107 Days	
	Before denoising	After denoising	Before denoising	After denoising	Before denoising	After denoising
# Nodes	9004	8872	9710	9336	10,956	9904
# Edges	68,683	53,815	127,205	56,685	306,062	60,757
# Paths	27,179	19,176	43,944	20,010	109,568	21,440

**Table 9:** Comparison of the ranking results based on the data collected in 5 days, 40 days and 107 days.

		5 Days	40 Days	107 Days
Nodes of Top-20	$B_{N}$	202.97.21.49; 202.97.21.57	202.97.21.49; 202.97.21.57	202.97.21.49; 202.97.21.57
		202.97.21.45; 202.97.21.53	202.97.21.45; 202.97.21.53	202.97.21.45; 202.97.21.53
		202.97.33.110; 202.97.4.98	202.97.33.110; 202.97.4.98	202.97.33.110; 202.97.4.98
		202.97.19.154; 202.97.23.114	202.97.19.154; 202.97.23.114	202.97.19.154; 202.97.23.114
		202.97.23.118; 202.97.23.110	202.97.23.118; 202.97.23.110	202.97.23.118; 202.97.23.110
	$B_{P}$	118.123.230.113	118.123.230.113; 118.123.230.21	118.123.230.85; 118.123.230.37
		118.123.230.85	118.123.230.121; 118.123.230.85	118.123.230.201; 118.123.230.121
		118.123.230.201	118.123.230.201; 118.123.230.205	118.123.230.205; 118.123.230.21
		118.123.230.205	118.123.230.37	118.123.230.113
	Other nodes	118.112.255.49; 118.112.255.33	118.112.255.49	118.112.255.49
		118.112.255.53; 110.188.6.6	118.112.255.33	118.112.255.33
		118.112.255.37; 182.140.220.93	118.112.255.69	118.112.255.69

As can be seen from Table 8, the number of nodes, edges and paths in the data of 107 days is 1.12, 2.41 and 2.49 times of 40 days, 1.22, 4.47 and 4.03 times of 5 days, respectively. From Table 9, we can see that in the 5-day results, 10 national-level backbone nodes and 4 provincial-level backbone nodes are found; in the 40-day results, 10 national-level backbone nodes and 7 provincial-level backbone nodes are found; in the 107-day results, 10 national-level backbone nodes and 7 provincial-level backbone nodes are found.

This shows that in the case of a large difference in measurement duration, the number of paths, edges, and nodes have significant changes in the obtained topology graph. However, the data scale after denoising does not change much, as well as the vital nodes discovery results of the proposed algorithm. At the same time, when the measurement duration is short, it is impossible to find enough vital nodes because the number of stable paths is insufficient. Therefore, it is necessary to mine the vital nodes after the data collection reaches a certain scale. When the number of stable paths is sufficient, the proposed algorithm can discover all the vital nodes that can be mined under this vantage point.

#### Comparison of experimental results before and after denoising

**Figure 7:**
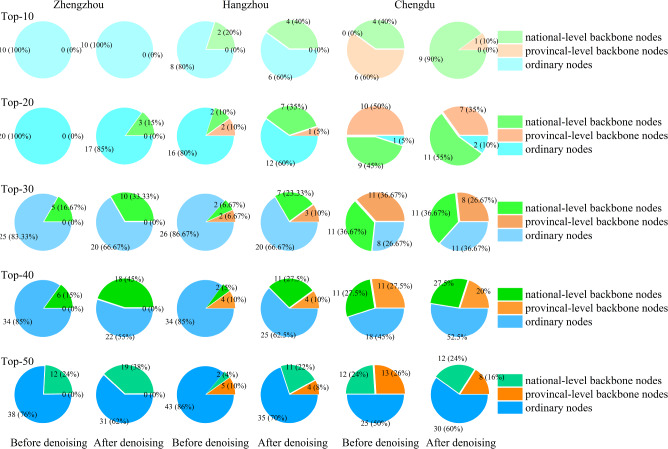
Results before and after denoising.

This section compares the scale of networks and ranking accuracy before and after denoising, and the results are shown in Table 10 and Fig. 7.

**Table 10:** Comparison of the network scale and ranking results before and after denoising.

		Zhengzhou		Hangzhou		Chengdu	
		Before denoising	After denoising	Before denoising	After denoising	Before denoising	After denoising
Scale of network	# Nodes	7331	6785	20,401	19,317	10,956	9904
	# Edges	208,971	38,604	670,050	134,954	306,062	60,757
	# Paths	76,014	15,166	248,298	47,979	109,568	21,440
Top-10	$B_{N}$	0	0	2	**4**	4	**9**
	$B_{P}$	0	0	0	0	**6**	1
	*B*	0	0	2	**4**	10	**10**
Top-20	$B_{N}$	0	**3**	2	**7**	9	**11**
	$B_{P}$	0	0	**2**	1	**10**	7
	*B*	0	**3**	4	**8**	**19**	18
Top-30	$B_{N}$	5	**10**	2	**7**	**11**	**11**
	$B_{P}$	0	0	2	**3**	**11**	8
	*B*	5	**10**	4	**10**	**22**	19
Top-40	$B_{N}$	6	**18**	2	**11**	**11**	**11**
	$B_{P}$	0	0	4	**4**	**11**	8
	*B*	6	**18**	6	**15**	**22**	19
Top-50	$B_{N}$	12	**19**	2	**11**	**12**	**12**
	$B_{P}$	0	0	**5**	4	**13**	8
	*B*	12	**19**	7	**15**	**25**	20

Significant values are in bold.

Table 10 shows the network scale before and after denoising, including the number of nodes, the number of edges, and the number of paths in the network topology. $B_{N}$/$B_{P}$/*B* respectively represent the number of national backbone node / provincial backbone node / backbone nodes among the top-k nodes in the ranking results. As can be seen from Table 10, denoising alternative paths can reduce about 7% of nodes, 80% of edges, and 80% of paths, which significantly reduces the scale of data processing. In addition, the bold number indicates the larger value in the comparison result before and after denoising. We can see that in a total of 15 groups of comparisons in 3 cities, the ranking metric after denoising (i.e., RWDC) performs better in 10 groups.

The green/orange/blue sectors in Fig. 7 respectively represent the number of national-level backbone nodes / provincial-level backbone nodes / backbone nodes among the top-k nodes in the ranking results. It can be seen that, in most cases, the results of the proposed algorithm have larger green area and smaller blue area, indicating that the proposed algorithm can find more (or the same) number of national-level backbone nodes than that of before denoising.

Combining the results in Table 10 and Fig. 7, it can be concluded that the proposed topology denoising method can significantly improve the accuracy of the ranking result and reduce the scale of data processing.

#### Comparison of the proposed algorithm and baseline algorithms

**Figure 8:**
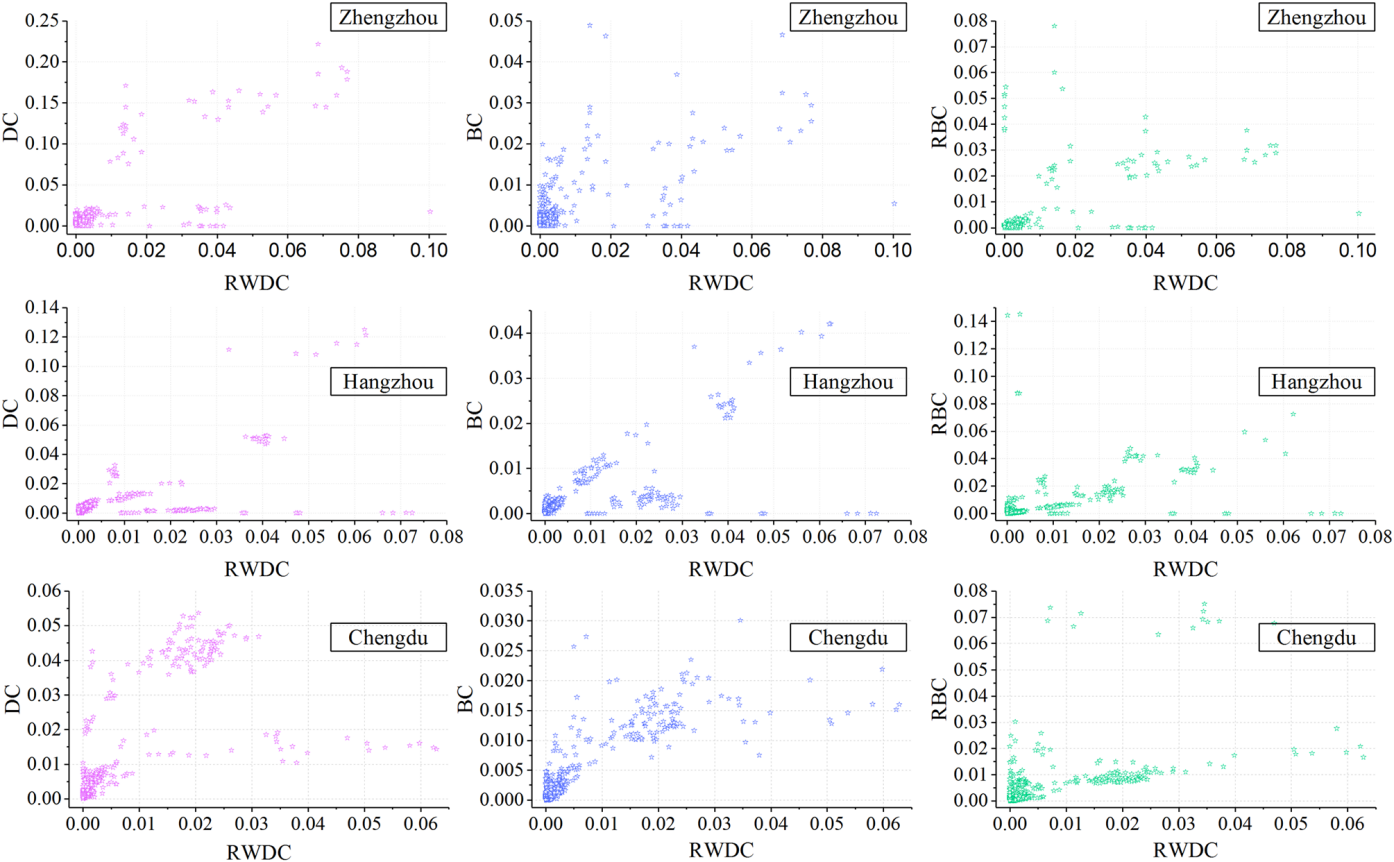
Corresponding relationships between RWDC and DC, BC, RBC.

To validate the effectiveness of the proposed algorithm, the result obtained by RWDC is compared with those of DC, BC and RBC. The corresponding relationship between RWDC and DC, BC, RBC are shown in Fig. 8.

The abscissa in Fig. 8 represents the node ranking result calculated by the proposed algorithm, and the ordinate represents the node ranking result calculated by the baseline algorithms. It can be seen that the results of the 4 metrics are quite different. The vital nodes in the ranking results of DC, BC, and RBC do not always rank high in RWDC.

**Figure 9:**
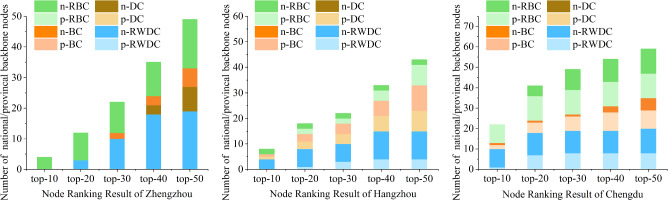
Comparison of the ranking results of the 4 metrics.

To compare the accuracy of the 4 metrics, this section uses existing public databases to verify the results. The comparison results are shown in Table 11 and Fig. 9.

**Table 11:** Comparison between the proposed algorithm and baseline algorithms.

		Zhengzhou				Hangzhou				Chengdu			
		RWDC	DC	BC	RBC	RWDC	DC	BC	RBC	RWDC	DC	BC	RBC
Top-10	$B_{N}$	0	0	0	**4**	**4**	0	0	2	**9**	0	1	0
	$B_{P}$	0	0	0	0	0	**1**	**1**	0	1	0	2	**9**
	*B*	0	0	0	**4**	**4**	1	1	2	**10**	0	3	9
Top-20	$B_{N}$	3	0	0	**9**	**7**	0	0	2	**11**	0	1	5
	$B_{P}$	0	0	0	0	1	**3**	**3**	2	7	0	5	**12**
	*B*	3	0	0	**9**	**8**	3	3	4	**18**	0	6	17
Top-30	$B_{N}$	**10**	0	2	**10**	**7**	0	0	2	**11**	0	1	10
	$B_{P}$	0	0	0	0	3	**4**	**4**	2	8	0	7	**12**
	*B*	**10**	0	2	**10**	**10**	4	5	4	19	0	8	**22**
Top-40	$B_{N}$	**18**	3	3	11	**11**	0	0	2	**11**	0	3	**11**
	$B_{P}$	0	0	0	0	4	**6**	**6**	4	8	0	9	**12**
	*B*	**18**	3	3	11	**15**	6	6	6	19	0	12	**23**
Top-50	$B_{N}$	**19**	8	6	16	**11**	0	0	2	**12**	0	6	**12**
	$B_{P}$	0	0	0	0	4	8	**10**	8	8	0	9	**12**
	*B*	**19**	8	6	16	**15**	8	10	10	20	0	15	**24**

Significant values are in bold.

In Table 11, Top-k represents the number of backbone nodes in the top k nodes obtained by various algorithms, and the value bolded in the table is the maximum value of the number of backbone nodes found by the 4 algorithms. In Fig. 11, the green/orange/gray/blue cylindrical represents the experimental results of RBC/BC/DC/RWDC, respectively; the light bars represent the number of provincial-level backbone nodes, and the dark bars represent the number of national-level backbone nodes.

Taking the results of Hangzhou as an example, it can be seen from Fig. 9 and Table 11 that among the top-10/20/30/40/50 nodes obtained by various algorithms, the proposed algorithm can find the largest number of national-level and provincial-level backbone nodes. Besides, among 15 groups of comparison in 3 cities, the proposed algorithm finds more (or the same number) backbone nodes in 10 groups, and finds more (or the same number) national-level backbone nodes in 13 groups. It comes to a conclusion that the proposed algorithm can find more vital nodes than DC, BC and RBC.

Take the experimental results in Chengdu as an example, the top-10 nodes and validation results under the 4 metrics are shown in Table 12.

**Table 12:** Comparison of top-10 nodes ranked by RWDC, DC, BC and RBC in Chengdu.

Rank	RWDC	Label	DC	Label	BC	Label	RBC	Label
1	202.97.21.49	$B_{N}$	171.208.199.254	$\times$	118.123.230.121	$B_{P}$	118.123.230.121	$B_{P}$
2	202.97.21.57	$B_{N}$	61.139.121.70	$\times$	118.123.230.41	$B_{P}$	118.123.230.41	$B_{P}$
3	202.97.21.45	$B_{N}$	171.208.196.14	$\times$	182.140.220.241	$\times$	118.123.230.205	$B_{P}$
4	202.97.21.53	$B_{N}$	61.139.121.74	$\times$	118.112.255.37	$\times$	118.123.230.25	$B_{P}$
5	202.97.33.110	$B_{N}$	110.188.6.86	$\times$	202.97.21.45	$B_{N}$	118.123.230.21	$B_{P}$
6	202.97.4.98	$B_{N}$	118.112.255.53	$\times$	118.112.255.65	$\times$	118.123.230.49	$B_{P}$
7	202.97.19.154	$B_{N}$	118.112.255.37	$\times$	182.140.220.93	$\times$	118.123.230.37	$B_{P}$
8	118.123.230.85	$B_{P}$	171.208.199.238	$\times$	182.140.220.109	$\times$	118.123.230.201	$B_{P}$
9	202.97.23.114	$B_{N}$	110.188.6.102	$\times$	118.112.255.69	$\times$	118.123.230.85	$B_{P}$
10	202.97.23.118	$B_{N}$	110.188.6.70	$\times$	118.112.255.45	$\times$	118.123.230.117	$B_{P}$

Significant values are in bold.

According to Table 12, the proposed algorithm discovers 9 national-level backbone nodes and 1 provincial-level backbone node in the top 10 nodes. While DC finds no backbone node in the top 10 nodes. BC discovers 1 national-level backbone node and 4 provincial-level backbone nodes; RBC discovers none national-level backbone node and 10 provincial-level backbone nodes.

The experimental results demonstrate that the proposed algorithm can find more backbone nodes than DC, BC and RBC, and the results are more accurate.

## Conclusion

This manuscript proposes an algorithm for discovering vital nodes in regional networks based on stable path analysis. The network topology denoising method based on stable paths proposed by this algorithm can effectively reduce the scale of processed data, and the edge-weighting method based on routing characteristics can significantly distinguish the role of edges in actual communication. Experimental results show that, the proposed algorithm can find more vital nodes than existing algorithms. However, due to the impact of load balancing and the limitation of the deployment of vantage points, this algorithm cannot find all the vital nodes in the target area. This is determined by stable paths passed by the experimentally deployed vantage points. For this reason, we will study how to deploy vantage points to obtain a relatively complete regional network topology in future work, then improve the discovery ability of vital nodes in the target area.

## Data Availability

The datasets generated and analyzed during the current study are not publicly available due to the security and privacy of network facilities, but are available from the corresponding author on reasonable request. Meanwhile, the six IP address databases used in this manuscript are available at: http://www.maxmind.com/, http://www.ip2location.com/ , http://www.whois.com/, http://www.ipip.net/, https://www.ipplus360.com/, http://www.ip.cn/.
